# The Deadly Doctor

**Published:** 1894-06

**Authors:** 


### EDITORIAL DEPARTMENT.

F. E. DANIEL, M. D., Editor.
S. E. HUDSON, M. D., Managing Editor.
A. J. SMITH. M. D., Galveston, Associate Editor.

EDITORIAL STAFF:

PROF. J. E. THOMPSON, M. D., Texas Medical College, Galveston; Surgery.
PROF. WM. KEILLER, M. D., Texas Medical College, Galveston; Obstetrics and Gynecology.
PROF. DAVID CERNA, M. D., Texas Medical College, Galveston; Therapeutics.
PROF. A. J. SMITH, M. D., Texas Medical College, Galveston; Medicine.
DR. ISADORE DYER, Tulane University; Dermatology.
DR. R. H. L. BIBB, Saltillo, Mexico; Foreign Correspondent.
DR. ROBT. MORRIS, Charity Hospital, N. O.; Clinical Reports

### THE DEADLY "DOCTOR."

"Children and fools should beware of sharp tools" is an old saying, of much significance. It has a wide application. An instance wherein the injunction was unheeded by one who may well be classed with the latter, has come to the knowledge of the JOURNAL. The adage may be paraphrased thus: "ignorant doctors should beware of dangerous drugs."

Attending a large female school in South Texas is a certain young girl from a neighboring county. She suffered much from headache, and so wrote her mother. The child is thirteen years of age. The mother consulted her "family physician," and said f. p. sent the child a box of 12 powders, with the following lucid directions written on the lid:

"Directions: Take 5 powders (every 3 hours one dissolve in a tablespoonful of water during twelve hours."

Thinking, from the wording of the directions, that she was to take five powders at one time, and the powders being very large, —five grains each—the child tried to make matters satisfactory by taking them—according to "directions" also—one, dissolved in tablespoonful of water, one at a time, every few minutes, till she had taken five. She thus took five powers in five doses all within about ten minutes.

She went to her room where soon afterwards she was discovered lying across the bed—to all appearance dead. Dr. Jones, living near the college, was summoned, and found the child deeply cyanosed, respiration almost stopped—sighing occasionally, only, lips livid, pupils dilated, skin cold, clammy and almost insensible to touch,—limbs paralyzed,—still she said she felt well, and was surprised at the attention being given her. Prompt measures restored the child in due time.

She had taken twenty-five grains antifebrin,--and by direction of her mother's "family physician." Had she been able, she would have taken the 60 grains in 12 hours,—and been a corpse.

